# Insights on glicentin, a promising peptide of the proglucagon family

**DOI:** 10.11613/BM.2017.034

**Published:** 2017-06-15

**Authors:** Juliette Raffort, Fabien Lareyre, Damien Massalou, Patrick Fénichel, Patricia Panaïa-Ferrari, Giulia Chinetti

**Affiliations:** 1 Clinical Chemistry Laboratory, University Hospital of Nice, Nice, France; 2 Université Côte d’Azur, Institute for Research on Cancer and Aging, Nice, France; 3 Department of Vascular Surgery, University Hospital of Nice, Nice, France; 4 Department of General Surgery and Digestive Cancerology, University Hospital of Nice, Nice, France; 5 Department of Endocrinology, University Hospital of Nice, Nice, France

**Keywords:** glicentin, proglucagon, glucagon-like peptide, oxyntomodulin, enteroendocrine cells

## Abstract

Glicentin is a proglucagon-derived peptide mainly produced in the L-intestinal cells. While the roles of other members of the proglucagon family including glucagon-like peptide 1, glucagon-like peptide 2 and oxyntomodulin has been well studied, the functions and variation of glicentin in human are not fully understood. Experimental and clinical studies have highlighted its role in both intestinal physiology and glucose metabolism, pointing to its potential interest in a wide range of pathological states including gastrointestinal and metabolic disorders. Due to its structure presenting many similarities with the other proglucagon-derived peptides, its measurement is technically challenging. The recent commercialization of specific detection methods has offered new opportunities to go further in the understanding of glicentin physiology. Here we summarize the current knowledge on glicentin biogenesis and physiological roles. In the limelight of clinical studies investigating glicentin variation in human, we discuss future directions for potential applications in clinical practice.

## Introduction

Gastrointestinal hormones correspond to a large family of various peptides released throughout the digestive tract including the stomach, the small intestine, the bowel as well as the pancreas (*1*-*4*). Thanks to their properties, these hormones play key roles in a wide range of physiological processes including control of appetite, regulation of glucose and lipid metabolism, digestive motility, secretion or trophicity. Interestingly, some hormones derive from larger precursors called prohormones. Prohormones have minimal hormonal effects and usually represent an inactivated form of hormones, ready to be activated by post-translational modifications.

Proglucagon is the archetype of a prohormone whose post-translational processing provides various peptides with distinct and complementary biological functions. While the variations and roles of some members of the proglucagon family such as glucagon-like peptide 1 (GLP-1), glucagon-like peptide 2 (GLP-2) and oxyntomodulin are well documented, glicentin has been poorly investigated so far. Development of commercialized detection methods has recently offered new opportunities to get further in understanding the biology of this member of the proglucagon family. Here, we summarize the current knowledge on glicentin roles and pathophysiological variations. In the limelight of clinical studies, we discuss potential applications and future directions for clinical research and medical practice.

## Biogenesis of glicentin

**Figure 1:**
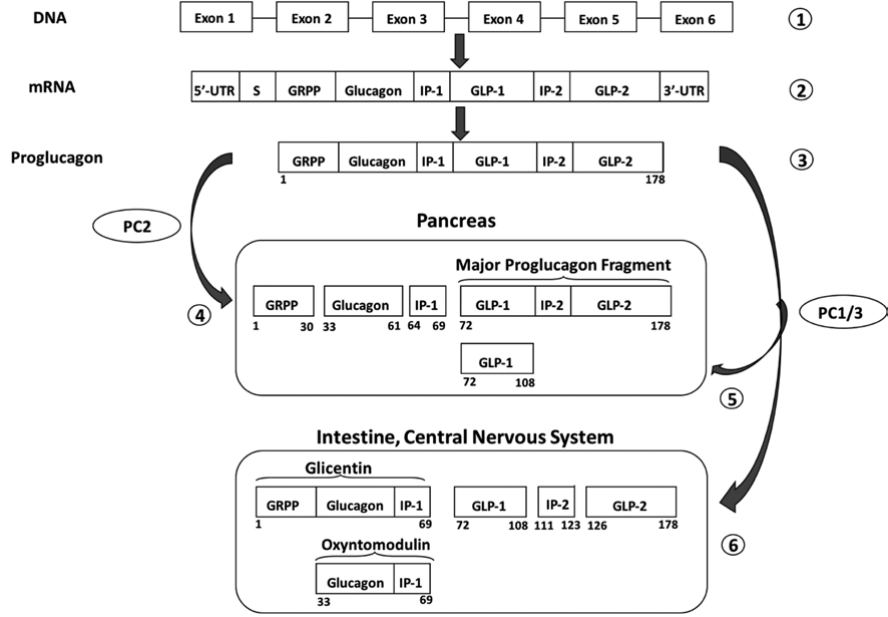
Processing of the proglucagon gene into proglucagon-derived peptides (modified from Baggio *et al.* (*4*), Holst *et al.* (*5*), Bataille *et al.* (*6*, *7*), Whiting *et al.* (*8*)) and DeFronzo *et al.* (*47*)). (*1*) The proglucagon gene is located on the chromosome 2 and is composed of 6 exons and 5 introns. (*2*) The gene transcription leads to a messenger RNA. (*3*) The messenger RNA (mRNA) is translated into the proglucagon, a 178 amino-acid precursor protein. (*4*) Posttranslational processing in the alpha pancreatic cells involves mainly the proconvertase 2 (PC2) and leads to the glicentin related pancreatic polypeptide (GRPP), the glucagon, the intervening peptide-1 (IP-1) and the major proglucagon fragment (MPF). (*5*) An alternative pathway involving proconvertase 1 and 3 (PC1/3) can lead to glucagon like peptide 1 (GLP-1) formation in the pancreas. (*6*) In the enteroendocrine L-cells and in the central nervous system, post-translational processing of proglucagon is mediated by proconvertases 1 and 3 and liberates the glicentin, the oxyntomodulin, the glucagon like peptide 1, the Intervening Peptide-2 (IP-2) and the glucagon like peptide 2 (GLP-2). DNA - deoxyribonucleic acid.

Glicentin belongs to the proglucagon-derived peptides. The proglucagon gene is mainly expressed in the alpha-cells of the endocrine pancreas, as well as the intestinal L-cells (*4*, *5*). The proglucagon gene is located on the chromosome 2 and is composed of 6 exons and 5 introns (Figure 1). The gene transcription leads to a messenger RNA (mRNA) which is translated into proglucagon, a 178 amino-acid precursor protein. While the structure of the proglucagon is identical in the pancreas and the intestine, its post-translational processing differs between these two organs, leading to various peptides, with distinct and complementary biological functions.

Schematically, the maturation in the pancreas involves the proconvertase 2 and leads to four main products: the glicentin related pancreatic polypeptide (GRPP), the glucagon, the intervening peptide-1 (IP-1) and the major proglucagon fragment (MPF) (*4*-*8*). In the enteroendocrine L-cells, post-translational processing of proglucagon, mainly mediated by proconvertases 1 and 3, liberates the glicentin, the oxyntomodulin, the GLP-1, the intervening peptide-2 (IP-2) and the GLP-2 (*4*, *6*-*9*). The mature form of glicentin is composed of 69 amino acids and contains the entire sequences of glucagon and oxyntomodulin (*10*).

As the enteroendocrine L-cells are present from duodenum to rectum, glicentin can potentially be synthetized all along the digestive tract. Nevertheless, L-cells are rare before the terminal ileum and experiments in animal models revealed that tissue concentration of oxyntomodulin/glicentin were higher in distal ileum, caecum, and proximal colon compared to duodenum and proximal jejunum (*11*, *12*). These results suggest that the distal small bowel and the proximal colon are the main sites of the glicentin synthesis.

Nevertheless, recent advances in the study of molecular mechanisms have unravelled previously unsuspected pathways. Indeed, several studies have highlighted that alpha pancreatic cells express proconvertase 1 and 3 and are able to produce and secrete GLP-1 (*13*, *14*). Further, an immature form of glicentin has been identified in the alpha cells (*6*). Interestingly, the proglucagon gene is also expressed by specific neurons in the central nervous system. Its post-translational processing is similar to the intestine and mature glicentin thus has been identified in the central nervous system (*6*). Hence, it is not excluded that future research on glicentin biogenesis could reveal new sites and pathways involved in its production.

## Glicentin and intestinal physiology

Experimental studies in several animal models including piglets, dogs and rats have reported the role of glicentin in intestinal physiology (Table 1).

**Table 1:** Summary of experimental studies on glicentin functions and variation

**Functions**	**Models**	**Aim**	**Main results**	**References**
**Factors stimulating glicentin secretion**	Piglets - administration of butter, glycerol and sodium palmitate into the duodenum	Investigate the response of plasma glicentin to intraluminal fat loading.	Increase of plasma glicentin following butter and palmitate administration Slight increase of plasma glicentin, but not significant following glycerol administration	Ohneda *et al*. (*15*)
	Piglets - administration of a mixture of 10 amino acids into the duodenum Dogs - pancreatectomy, administration of arginine	Explore the effect of amino acids on glicentin secretion.	Increase of plasma glicentin following amino acids administration Increase of total immunoreactive glucagon in pancreatectomized dogs following amino acids administration Administration of amino acids enhances glicentin secretion from the gut.	Ohneda *et al.* (*16*)
	Piglets - administration of glucose into the duodenum	Investigate the secretion of glicentin in response to intraluminal administration of glucose.	Increase of plasma glicentin after glucose loading	Ohneda *et al.* (*17*)
**Effect of glicentin on intestinal trophicity**	Rats - subcutaneous injection of glicentin (50 µg/kg every 12h for 2 weeks) In vitro - intestinal epithelial cell line IEC-6, incubation with glicentin (0–1000 ng/mL) for 48h	Examine the trophic effects of glicentin on small-intestinal mucosa and on the small-intestinal epithelial cell line IEC-6	Jejunum - increase of weight, protein, DNA content and alkaline phosphatase activity in animals who were injected with glicentin; increase of ornithine decarboxylase activity after intraperitoneal injection of glicentin Ileum - no difference between animals injected with glicentin and the control group *In vitro* - increase of tritium-thymidine incorporation and the number of IEC-6 cells in presence of glicentin Trophic effect of glicentin on intestinal mucosa.	Myojo *et al.* (*18*)
	Rats - parenteral nutrition for 6 days and administration of 1, 4, 20 or 80 µg/rat of glicentin via the jugular vein	Examine the effects of glicentin on intestinal proliferation *in vivo* in the rat.	Dose-dependent increase of mucosal proliferation in the small intestine induced by glicentin, with an effect more pronounced in the ileum Decreased crypt fission of the ileum in the 20 and 80 µg glicentin groups compared to controls No significant effect of glicentin on proliferation or fission in the colon Trophic effect of glicentin on the small intestine but not on the colon.	Sasaki *et al*. (*19*)
	Rats - resection of the small intestine and aministration of methionyl rat glicentin (20 μ/day/rat) from the second day after the operation and during 28 days using miniosmotic pumps	Explore the effects of glicentin on intestinal adaptive responses to 70% resection of small intestine in rats	Rats with 70% distal intestinal resection - significant increase of the weight of the residual duodenum and its mucosal weight, protein, and diamine oxydase activity compared to controls. No significant change on the residual jejunum. Rats with 70% proximal intestinal resection - significant increase of the weight of the residual duodenum and its mucosal weight, protein, and diamine oxydase activity compared to controls Stimulating activity of glicentin on the adaptive responses to massive intestinal resection.	Hirotani *et al.* (*23*)
	Rats - construction of loops of jejunum and ileal Thiry-Vella fistulas (TVFs) that were isolated from the luminal stream and subcutaneous injection of glicentin (50 µg/kg every 12 hours for 1 week)	Determine whether the trophic effect of glicentin is mediated by mechanism involving luminal or non-luminal factors and determine whether glicentin exerts a differential trophic effect on jejunal or ileal mucosa	Effect of glicentin in the jejunal TVF group - increase of mucosal growth measurements in the TVF and the intact jejunum Effects of glicentin on the proximal gut mucosa may be caused by a combination of non-luminal and luminal factors. Effect of glicentin in the ileal TVF group - stimulation of the proliferation of intact ileal mucosa but no effect on the ileal TVF Effect on the ileal mucosa may be influenced by luminal contents and endogenous secretions. Differential trophic effect of glicentin on mucosa of the proximal jejunum and distal ileum.	Hashimoto *et al.* (*24*)
**Effect of glicentin on bacterial internalization and *Helicobacter pylori* infection**	Cell line, *in vitro* INT-407 cell lines - incubation of glicentin (100 ng/ml to 1 µg/ml) for 24h Assays on bacterial internalization with *Salmonella enteritidis* IFO 3313, *Escherichia coli* ATCC M44 and *Enterococcus faecalis* ATCC 29212	Determine the effect of recombinant human glicentin on bacterial internalization by confluent enterocytes	Lower bacterial internalization through the glicentin-treated enterocytes than the non-treated Dose dependent effect of glicentin on bacterial internalization in the range from 500 to 1 µg/mL Glicentin acts as a barrier-sustaining agent that inhibits extra-intestinal invasion of enteric bacteria and decreased bacterial translocation.	Chiba *et al*. (*25*)
	Human gastric biopsies - 7 cases with endoscopical intestinal metaplasia; 47 cases with no endoscopical intestinal metaplasia 18 biopsies with histological intestinal metaplasia 29 biopsies with no intestinal metaplasia	Investigate the relationship between *Helicobacter pylori* infection, intestinal metaplasia and glicentin expression in the gastric mucosa	Detection of mRNA glicentin expression in 100% of cases with endoscopical intestinal metaplasia, in 76,7% of cases with no endoscopical intestinal metaplasia Glicentin mRNA expression statistically associated with the existence of histological intestinal metaplasia and positively correlated with *Helicobacter pylori* infection	Ishihara *et al.* (*33*)
**Effect of glicentin on gastro-intestinal motility**	Rats - glicentin IV infusion during the 5 min preceding food onset and during the first 15 min of food intake	Investigate the effect of a systemic increase of glicentin on food intake, postprandial myoelectrical activity in duodenum, jejunum and ileum and the oro-caecal transit	No effect of systemic injection of glicentin on food intake Reduction of duration of the postprandial myoelectrical activity on duodenum and jejunum induced by glicentin	Pellissier *et al.* (*26*)
	Dogs - use of vagally denervated gastric pouches equipped with four strain gauge force transducers on the pouch, gastric body, antrum and duodenum. Injection of glicentin intra-veinously (1 nmol/kg)	Explore the effect of glucagon, GLP-1, GLP-2 and glicentin on gastroduodenal motility and their mechanisms of action	Glicentin did not affect motilin-induced phase III contractions at any site Glucagon inhibited contractions in the pouch and the stomach GLP-1 inhibited contractions at all sites GLP-2 inhibited contractions in the pouch Differential effect of proglucagon-related peptides on gastroduodenal motility.	Shibata *et al.* (*48*)
	Rabbit, *in vitro* - isolation of smooth muscle cells (SMCs) from rabbit antrum, incubation with glicentin (5 nmol/L)	Explore the effect of glicentin SMCs contraction isolated from the rabbit antrum	Induction of SMCs contraction by glicentin Maximal contraction at 30 s	Rodier *et al.* (*29*)
	Rabbit, *in vitro* - isolation of SMCs from rabbit antrum, incubation with glicentin (2 nmol/L)	Describe the morphological and functional characteristics of a model of cultured digestive SMCs exhibiting a differentiated phenotype	Induction of SMCs contraction by glicentin	Jarousse *et al.* (*30*)
**Effect of glicentin on gastro-intestinal motility**	Rabbit, *in vitro* - isolation of SMCs from rabbit antrum, incubation with glicentin (0.3 nmol/L)	Investigate the modifications of the calcium/ phosphoinositide and the cyclic adenosine monophosphate (cAMP) pathways in rabbit antral SMCs	Induction of SMCs contractions by glicentin Induction of a biphasic and rapid inositol (*1*, *4*, *5*)-trisphosphate (Ins(1,4,5)P3) production by glicentin In the absence of extracellular calcium: glicentin induced Ins(1,4,5)P3 production became monophasic Induction of a rapid increase of intracellular free calcium induced by glicentin Reduction of the cAMP content by glicentin of cells stimulated by forskolin Contractile effect of glicentin potentially mediated by stimulation of the phosphoinositide hydrolysis and inhibition of cAMP production Action of glicentin potentially mediated by receptor coupled to G proteins	Rodier *et al.* (*28*)
	Human, *in vitro* - 32 preparations of human normal jejunal muscle strips	Investigate the enteric nervous responses to glicentin in the normal small bowel	Inhibition of contraction reaction by glicentin after blockade of the adrenergic and cholinergic nerve Regulating inhibition of the contraction reaction in normal human jejunum via non-adrenergic non-cholinergic (NANC) nerves, and has a direct action on the jejunal muscle receptor.	Tomita *et al.* (*27*)
	Human, *in vitro* patients who underwent resections for colon adenocarcinoma (8 right colons, 14 left colons); isolation of SMCs Rabbit - SMCs from antrum	Investigate the effect of glicentin on the motor activity of colon	Human SMCs from colon - dose-related contraction of SMCs induced by glicentin. Decrease of contractile activity induced by glicentin when incubated with exendin- (*9*–*39*), a selective antagonist of GLP-1 receptor Rabbit SMCs from antrum - exendin- (*9*–*39*) dose dependently reduced the contractile activity of glicentin Effect of glicentin in SMC contraction may be mediated by GLP-1 receptor.	Ayachi *et al.* (*31*)
**Effect of glicentin on gastric acid secretion**	Rats - use of chronic gastric fistulas, stimulation of gastric acid secretion by pentagastrin, injection of glicentin intra-veinously (bolus 3µg/kg, followed by 600 ng/h or 120 ng/h)	Investigate the role of glicentin on gastric acid secretion	Inhibition of gastric acid secretion by glicentin	Kirkegaard *et al.* (*32*)
**Effect of glicentin on glucose metabolism**	Rats, *in vitro* - isolation of hepatocytes	Study the effect of glicentin on the glucose production and cAMP accumulation of isolated hepatocytes	Glicentin and glucagon stimulated the release of glucose from hepatocytes into the medium Stimulation of cAMP production by glucagon and glicentin Degradation of glicentin into low molecular weight fragments during incubation with hepatocytes, with some fragments similar to glucagon	Thieden *et al.* (*35*)
	Dogs - use of *in vivo* local circulation in canine pancreas; administration of glicentin into the pancreaticoduodenal artery (400 ng for 10 min)	Investigate the effect of glicentin-related peptides on the endocrine pancreatic function	Plasmatic concentrations after glicentin (*1*-*16*) administration in the canine pancreas - decrease of glucagon Plasmatic concentrations after glicentin (62-69) administration in the canine pancreas - decrease of insulin and glucagon Glicentin released during nutrient intake might inhibit the secretion of glucagon.	Ohneda *et al.* (*36*)
	Dogs - use of *in vivo* local circulation in canine pancreas; administration of glicentin into the pancreaticoduodenal artery (200 pmol for 10 min)	Study the role of glicentin-related peptides on the endocrine pancreas	Plasmatic concentrations after glicentin (*1*-*16*) and glicentin (62-69) administration in the canine pancreas - increase of insulin and decrease of glucagon Plasmatic concentrations after glicentin (62-69) administration in the canine pancreas - increase of insulin and decrease of glucagon Maximum response of insulin and glucagon within 20 min after glicentin (*1*-*16*) and glicentin (62-69) administration - increase of insulin and decrease of glucagon	Ohneda *et al.* (*34*)
	Dogs - use of *in vivo* local circulation in canine pancreas; administration of glicentin into the pancreaticoduodenal artery (100 and 400 pmol for 10 min)	Explore the effect of human recombinant glicentin on the endocrine function of the pancreas	Plasmatic concentrations after glicentin administration at a dose of 100 pmol during glucose infusion - no significant change in blood glucose, slight increase of insulin, but not significant; no significant change of glucagon Plasmatic concentrations after glicentin administration at a dose of 400 pmol during glucose infusion - significant increase of blood glucose, significant increase of insulin, increase of glucagon Plasmatic concentrations after glicentin administration at a dose of 100 pmol during arginine infusion - no significant change in blood glucose, significant increase of insulin, no significant change of glucagon Plasmatic concentrations after glicentin administration at a dose of 400 pmol during arginine infusion - no significant change in blood glucose, significant increase of insulin, increase of glucagon Demonstration of the insulinotropic action of glicentin.	Ohneda *et al.* (*37*)
GLP 1/2 - glucagon like peptide 1 and 2.				

First, glicentin secretion is stimulated by food intake as revealed by increased plasma glicentin concentrations after glucose, lipids and amino-acids loading into the duodenum (*15*-*17*). Several studies in rat model have highlighted the trophic role of glicentin on intestinal mucosa. Indeed, an increase of several parameters of mucosal growth, including weight, protein and DNA content, alkaline phosphatase and ornithine decarboxylase activities have been reported in the jejunum after glicentin subcutaneous injection (*18*). *In vitro* studies confirmed the proliferative action of glicentin on epithelial cell line IEC-6. Another study showed the trophic effect induced by glicentin in the small intestine, with a more pronounced effect in the ileum (*19*), whereas Myojo *et al.* did not observe any effect of glicentin in the ileum (*18*). These heterogeneous results could be, at least partly, explained by differences in the methodology used including rat feeding, glicentin concentration and administration as well as markers used to assess intestinal proliferation. The authors further revealed a decrease of crypt fission in the ileum after glicentin administration (*19*). Crypt fission corresponds to the mechanism leading to new crypt formation and plays a major role in intestinal development. Increased crypt fission is observed following intestinal damage or ulceration (*20*), after administration of chemical carcinogens in rats and in humans with precancerous defects (*21*, *22*). Even if further investigations are required for a better understanding of the physiological significance of decreased crypt fission, these results pinpoint the potential role of glicentin in intestinal mucosa remodelling. The observation that glicentin is involved in mechanisms regulating mucosa development and renewal, has led some investigators to explore the effect of glicentin on adaptive response to intestinal resection (*23*). A significant increase of the weight of the residual duodenum and its mucosal weight, protein, and diamine oxidase activity was found in rats which underwent a 70% distal intestinal resection and which received glicentin after the intervention compared to control animals. These results underline the trophic role of glicentin in intestinal post-surgery remodelling. To go further into the physiological mechanisms, some authors aimed to determine whether the trophic effect of glicentin was mediated through pathways involving luminal or non-luminal factors (*24*). To achieve this goal, they used a model of rats with a construction of loops of jejunum and ileal fistulas that were isolated from the luminal stream. The authors found an increase of mucosal growth measurements in both the jejunal fistula and the intact jejunum, suggesting that the effect of glicentin on the proximal gut mucosa may be caused by a combination of non-luminal and luminal factors. In contrast, glicentin stimulated the proliferation of intact ileal mucosa but had no effect in the ileal fistula, meaning that the effect of glicentin on the ileal mucosa may be influenced by luminal content and endogenous secretions. Taken together, these results suggest a differential trophic effect of glicentin on mucosa on the proximal jejunum and distal ileum. In addition to its trophic effect on intestinal mucosa, glicentin may also act as a barrier-sustaining agent. Indeed, the effect of recombinant human glicentin on bacterial internalization by confluent INT407 enterocytes cell lines using *Salmonella enteritidis*, *Escherichia coli* and *Enterococcus faecalis* was determined (*25*). A lower bacterial internalization through the glicentin-treated enterocytes was observed compared to the non-treated cells, revealing the inhibitory effect of glicentin on bacterial translocation and intestinal invasion.

Intestinal motility represents a factor potentially influencing bacterial internalization. *In vivo* studies in animal models highlighted the effect of glicentin on gut motility, as demonstrated by the reduction of the duration of the postprandial myoelectrical activity on duodenum and jejunum in rats infused by glicentin (*26*). *Ex vivo* studies using preparation of human normal jejunal muscle strips further revealed the inhibitory effect of glicentin on contraction reaction after blockade of the adrenergic and cholinergic nerve (*27*). In contrast, studies on smooth muscle cells (SMCs) isolated from rabbit antrum revealed the stimulatory action of glicentin on muscle contraction (*28*-*30*). This contractile effect of glicentin may be potentially mediated by receptor coupled to G proteins and through the stimulation of the phosphoinositide hydrolysis and cyclic adenosine monophosphate (cAMP) production (*28*). Further, a reduced contractile activity of glicentin was observed after incubation with exendin- (*9*-*39*), a selective antagonist of GLP-1 receptor, suggesting that glicentin action may be relayed, at least partly, through GLP-1 receptor (*31*). Similarly to what was observed using SMCs from rabbit antrum, a dose-related contraction of SMCs isolated from human colon was found and this effect was inhibited after incubation with exendin- (*9*-*39*) (*31*). Taken together, these results reveal the stimulating role of glicentin on SMC contraction along the digestive tract, an effect which may involve GLP-1 receptor. At last, a study in rats revealed the inhibitory effect of glicentin on gastric acid secretion (*32*). Based on its action on the stomach, some investigators aimed at exploring the role of glicentin in metaplasia and its relationship with *Helicobacter pylori* infection (*33*). Using human gastric biopsies, they revealed that glicentin mRNA expression was associated with the existence of histological intestinal metaplasia and positively correlated with *Helicobacter* infection. Even if given its biological function, glicentin is of potential interest in the context of digestive oncology, these results should be interpreted with caution. In this study, mRNA glicentin expression was assessed, which means that the expression of the precursor proglucagon and not the mature glicentin form was analysed. Further investigations on glicentin protein expression would be required for a better understanding of its role and variation in context of intestinal metaplasia. To summarize, glicentin plays paracrine functions on the digestive tract through its involvement in various processes including regulation of intestinal trophicity and motility, as well as gastric acid secretion. Its potential implication in various pathophysiological conditions including cancers, infectious or inflammatory diseases remains to be explored.

## Glicentin and glucose metabolism

The family of proglucagon-derived peptides plays a major metabolic role through their involvement in glucose homeostasis. Glucagon is well known for its hyperglycemic action (*6*). On the opposite, main proglucagon-derived hormones produced in the intestine indirectly balance the effect of glucagon by exerting hypoglycemic actions. GLP-1 stimulates insulin secretion, improves insulin sensitivity and suppresses the release of glucagon (*3*, *7*). Oxyntomodulin has also a positive effect on insulin secretion (*2*, *3*). The role of GRPP in glucose metabolism has been less studied and is more controversial. Indeed, a few decades ago, Ohneda *et al.* observed an increase of plasma insulin concomitantly with a decrease of glucagon after GRPP administration into the pancreaticoduodenal artery in dogs, suggesting an incretin-like effect (*34*). However, a recent report revealed that GRPP inhibited glucose-stimulated insulin secretion from the isolated pancreas of adult male rats (*8*). The discrepancy between these two studies could be, at least partly, explained by differences in methodology used including species studied, GRPP synthesis and administration or pancreas preparation. This underlines the real need of further research to fully understand the specific role of each member of the proglucagon family.

In that context, glicentin effect was also investigated (Table 1). A first study found that glicentin stimulated the release of glucose from rat hepatocytes, with a stimulation of cAMP production, an effect comparable to what observed after incubation with glucagon (*35*). The authors observed that during incubation with hepatocytes glicentin was degraded into low molecular weight fragments, some being very similar to glucagon, addressing the question whether glicentin could exert glucagon-like effects through a possible degradation to glucagon. Nevertheless, these results should be interpreted with caution since this study was performed 30 years ago and since then the sequence defining glicentin protein as well as the technologies to identify and purify it have largely evolved. In fact, several relatively more recent *in vivo* studies in dogs demonstrated that administration of glicentin led to an increase of plasma insulin and a decrease of glucagon (*34*, *36*, *37*). Hence, similarly to GLP-1 and oxyntomodulin, glicentin has an insulinotropic action and exert an incretin-like effect. Even if the precise mechanisms involved in the insulin-releasing action of glicentin remains to be elucidated, these results pinpoint the potential interest of glicentin as marker and/or player of metabolic diseases such as diabetes or obesity.

## Circulating glicentin in humans

Given the role of glicentin in both intestinal physiology and glucose metabolism, its potential interest as non-invasive biomarker including digestive and metabolic diseases would be worth investigating. Due to its structure, assessment of circulating glicentin concentration is challenging to obtain a specific measurement which does not cross react with other proglucagon-derived peptides. Indeed, glicentin peptide contains the entire sequence of glucagon, GRPP and oxyntomodulin (Figure 1). This technical reason combined with the lack of commercialized methods to measure glicentin until recently contribute to explain why literature on circulating glicentin in human is poor and its variation not fully elucidated.

The first published reports investigated circulating glicentin concentration using a non-commercialized radioimmunoassay associated with chromatography (*38*) and a non-commercialized sandwich enzyme-linked immunosorbent assay (ELISA) (Table 2) (*39*-*41*). In the latest assay, the authors used two antibodies against the GRPP and the glucagon sequences and proved the specificity of the method to measure glicentin. Based on these studies, a solid phase two-site enzyme ELISA kit has recently been commercialized (Mercodia®, Uppsala, Sweden) and was used in several studies (*42*-*44*). The manufacturer tested the specificity of the assay and did not detect any cross reactivity with glucagon, oxyntomodulin, mini-glucagon, GLP-1 and GLP-2. At last, another commercialized ELISA kit has been developed (Merck-Millipore®, Billerica, United States) and was used in one study (*45*). The different ELISA tests share similarities, with detection limit for human glicentin close to 3 pmol/L, inter-assay variation less than 15% and intra-assay variation less than 10% (Table 2), allowing the comparison of results among different studies. However, data regarding pre-analytical conditions are to date extremely poor. According to results provided by manufacturers, higher glicentin concentrations were obtained when using dipeptidyl peptidase-4 inhibitor while protease and esterase inhibitors did not seem to have an effect on glicentin stability. Hence, when comparing results among different studies, it should be taken into consideration that methodology used for sample collection and preservation could have potentially impacted on glicentin concentrations.

**Table 2:** Methodology used to measure fasting circulating glicentin concentrations in human

**Detection method**	**Sample**	**Population**	**Glicentin concentration (pmol/L)**		**References**
**Median** **(interquartile** **range)**	**Mean** **± standard** **error mean**
Commercialized ELISA technique (Merck- Millipore®) - Inter-assay variation: < 15% - Intra-assay variation: < 10%	Plasma	Patients who had acute pancreatitis (N = 83)	6.2 (3.0 - 16.7)	-	Pendharkar *et al.* (*45*)
Commercialized ELISA technique (Mercodia®) - Detection limit: 3 pmol/L - Inter-assay variation: < 10.8% - Intra-assay variation: < 8%	Plasma	Lean adolescents (N = 19) Adolescents with obesity and normal glucose tolerance (N = 23) Adolescents with obesity and impaired glucose tolerance (N = 19) Adolescents with obesity and type 2 diabetes (N = 4)	17.6 (13 – 25) 23.6 (17.7 – 32.8) 18.2 (8.6 – 21.8) 15.1 (13.3 – 18)	-	Manell *et al.* (*42*)
	Serum	Lean adults (N = 52) Adults with severe or morbid obesity (N = 39)	24 (18 - 38) 12 (7.6 – 17)	-	Raffort *et al.* (*44*)
		Adults with severe or morbid obesity before Roux-en-Y Gastric Bypass (RYGB) surgery (N = 18) Adults 12 months after RYGB surgery (N = 18) Adults with severe or morbid obesity before Laparoscopic Sleeve Gastrectomy (LSG)† surgery (n= 12) Adults 12 months after LSG surgery (n= 12)	-	14 ± 3.6 19.7 ± 2.7 12.5 ± 1.4 16.4 ± 1.8	Raffort *et al.* (*43*)
Non-commercialized sandwich ELISA - Detection limit: 3.8 pmol/L - Inter-assay variation: < 8% - Intra-assay variation: < 5%	Plasma	Diabetic patients (N = 119) Controls (N = 6)	-	19.7 ± 2.1 18.6 ± 4.7	Naito *et al.* (*39*)
		Very-low-birthweight infants (N = 21)	NV	NV	Shimizu *et al.* (*41*)
		Children (N = 119)	NV	NV	Tadokoro *et al.* (*40*)
Non-commercialized radioimmunoassay and chromatography	Plasma	Non insulin-dependent diabetic patients (N = 8) Controls (N =8)	NV	NV	Orskov *et al.* (*38*)
RYGB: Roux-en-Y Gastric Bypass. LSG: Laparoscopic Sleeve Gastrectomy. NV - no exact values.					

The studies published so far confirmed the stimulating effect of glucose ingestion on glicentin secretion, as demonstrated by higher glicentin concentration after oral glucose tolerance test (OGTT) compared to fasting concentrations (*38*, *39*, *42*) (Table 3). Besides, similarly to what was observed in animal models, food intake stimulates glicentin secretion, as revealed by higher glicentin concentrations after feeding in children (*40*, *41*). As animal models demonstrated the role of glicentin in intestinal mucosa growth and trophicity, some authors investigated its variation in children from birth to 15 years’ age and revealed significant variations depending on children age (*40*). Interestingly, differences on fasting glicentin concentrations were observed between children with very low-birth-weight and normal birth weight children, suggesting that glicentin could play a role in gastrointestinal function and growth in the early period of life. In addition, feeding in children may shape and impact on glicentin secretion. Early enteral feeding within 24 hours after birth in very low birth weight children led to higher glicentin basal concentration at day 5–6 and day 14 after birth compared to infants fed more than 24 hours after birth (*41*). Besides, even if no significant difference in post-prandial glicentin concentrations was observed between breastfed and formula-fed children in this study, the potential impact of the nature of food ingested on glicentin secretion cannot totally be excluded.

**Table 3:** Summary of studies on circulating glicentin variation in human

**Population**	**Aim**	**Glicentin** **detection method**	**Main results**	**References**
- 83 patients who had acute pancreatitis: 30 developed abnormal glucose metabolism, 53 kept normal glucose metabolism	Explore the relationships between peptides known to be produced in both gut and brain and glucose metabolism in patients after acute pancreatitis	- Commercialized ELISA technique (Merck- Millipore®) - Plasma	- Significant decrease in glicentin, oxyntomodulin, vasoactive intestinal peptide (VIP) in individuals with abnormal glucose metabolism - Significant association between glicentin and secretin concentrations	Pendharkar *et al.* (*45*)
- 52 lean adults - 39 adults with severe or morbid obesity	Investigate serum glicentin concentrations during adult obesity and study its potential link with metabolic parameters	- Commercialized ELISA technique (Mercodia®) - Serum	- Significant decrease of glicentin concentration in patients with severe or morbid obesity compared to lean subjects - No linear correlation between glicentin concentration and body mass index, glycaemic parameters (glycaemia, insulinemia, C-peptide) or lipid parameters (total, HDL, LDL-cholesterol, triglyceride)	Raffort *et al.* (*44*)
- 30 adult patients with severe or morbid obesity, eligible to bariatric surgery: 18 patients had a Roux-en-Y Gastric Bypass (RYGB), 12 patients had a Laparoscopic Sleeve Gastrectomy (LSG), Follow-up at 3, 6 and 12 months post-surgery	Investigate fasting circulating glicentin variation in obese patients who underwent bariatric surgery	- Commercialized ELISA technique (Mercodia®) - Serum	- Significant increase of glicentin at 6 months post-surgery, with an effect more marked at 12 months - Tendency to have a more marked increase of glicentin after RYGB compared to LSG - Significant increase of glicentin/ glycaemia, glicentin/insulinemia, glicentin/C-peptide ratios after surgery - Improvement of metabolic parameters (anthropometric, glycaemic and lipidic) after surgery - No direct correlation between glicentin variation and metabolic parameters variation	Raffort *et al*. (*43*)
- 19 lean adolescents - 23 obese adolescents with normal glucose tolerance (NGT) - 19 obese adolescents with impaired glucose tolerance (IGT) - 4 obese adolescents with type 2 diabetes (T2D)	Explore fasting and postprandial plasma concentrations of the proglucagon- derived hormones (glucagon, glicentin, GLP-1) in adolescents with obesity	- Commercialized ELISA technique (Mercodia®) - Plasma	- No significant difference on fasting glicentin concentrations between lean adolescents and adolescents with obesity and NGT. - Lower fasting glicentin concentrations in adolescents with obesity and IGT compared to adolescents with obesity and NGT - Glicentin concentrations after oral glucose tolerance test (OGTT): lower in adolescents with obesity and IGT than those with NGT; peak at 30 minutes in lean adolescents and adolescents with obesity and NGT; peak at 15 minutes in adolescents with obesity and IGT;peak at 60 minutes in adolescents with obesity and T2D - Ratios of glicentin/glucagon and GLP-1/ glucagon: in fasting: lower ratios in obese adolescents with IGT and T2D than obese adolescents with NGT; during OGTT: lower ratios in obese adolescents with NGT than lean adolescents and lower ratios in obese adolescents with IGT and T2D than obese adolescents with NGT - Fasting plasma glicentin as a predictor of IGT in adolescents with obesity and normal fasting glucose: 100% sensitivity and 56% specificity	Manell *et al.* (*42*)
- 21 very-low-birthweight infants: 11 infants had early feeding with breast milk within 24h after birth, 10 infants had breast milk more than 24h after birth	Explore the effects of early enteral feedings and the secretion of gut hormones in very-low- birthweight infants	- Non- commercialized sandwich ELISA - Plasma	- Glicentin basal concentration: early feeding group: higher glicentin concentrations at day 5-6 and day 14 after birth than at day 1 – 2; control group: higher glicentin concentrations at day 14 after birth than at day 1 – 2; higher glicentin concentrations in the early feeding group compared to controls at day 5 - 6 and day 14 after birth - Post prandial glicentin concentration: early feeding group: higher glicentin concentrations after feeding than before feeding at day 5 - 6 and day 14; control group: higher glicentin concentrations after feeding than before feeding at day 14	Shimizu *et al.* (*41*)
- 119 developing children: 21 children aged 15 to 29 days, 16 children aged 1 to 5 months, 14 children aged 6 to 11 months, 16 children aged 1 to 3 years, 17 children aged 4 to 7 years, 18 children aged 8 to 11 years, 17 children aged 12 to 15 years - Term and preterm infants: 11 term and normal birthweight, 9 low birthweight, 10 very-low-birthweight - Normal birthweight children:14 breastfed and 11 formula-fed	Investigate the changes in basal plasma concentrations of glicentin in term and preterm developing children	- Non- commercialized sandwich ELISA - Plasma	- Glicentin basal concentration: higher glicentin concentrations in children aged 15 to 29 days, 1 to 5 months versus children aged 1 to 3 years, 4 to 7 years, 8 to 11 years; higher glicentin concentrations in children aged 6 to 11 months versus children aged 1 to 3 years, 8 to 11 years, 12 to 15 years; higher glicentin concentrations in very-low-birthweight children versus normal birthweight children at day 1 or 2 after birth - Post prandial glicentin concentration: higher glicentin concentrations after feeding than before at 1 - 2 days after birth and 5 - 6 days in normal and low-birth-weight children; higher glicentin concentrations after feeding than before at 14 days after birth in very low-birth-weight children; no significant difference in glicentin concentrations between breastfed and formula-fed group	Tadokoro *et al.* (*40*)
- 6 normal subjects - 119 diabetic patients - 15 gastrectomized patients - 9 with subtotal gastrectomy, 6 with total gastrectomy, 1 with massive small bowel resection	Explore plasma concentration of glicentin in diabetic and gastrectomized patients	- Non- commercialized sandwich ELISA - Plasma	- Fasting glicentin concentration: no significant difference on plasma glicentin concentrations between normal subjects and diabetics; no correlation between glicentin concentrations and plasma glucose - Glicentin concentration after OGTT: increase of plasma glicentin in normal subjects; higher peak of glicentin at 30 min in gastrectomized patients compared to normal subjects; no significant variation of glicentin in the patient who had a massive small bowel resection	Naito *et al.* (*39*)
- 8 noninsulin-dependent diabetics (T2D) - 8 weight-matched non-diabetic controls	Identify stimuli involved in the secretion of glicentin and investigate the impact of disturbance in glucose metabolism	- Non- commercialized radioimmunoassay and chromatography - Plasma	- Fasting glicentin: mean amount of total glucagon immunoreactivity eluting at 0.3 Kd (corresponding to the elution of porcine glicentin), lower in T2D than controls - Glicentin after OGTT: increase of glicentin secretion; mean amount of total glucagon immunoreactivity eluting at 0.3 Kd (corresponding to the elution of porcine glicentin) after OGTT: higher in T2D than controls	Orskov *et al.* (*38*)
VIP - vasoactive intestinal peptide. HDL - high-density lipoprotein. LDL - low-density lipoprotein. RYGB - Roux-en-Y gastric bypass. LSG - laparoscopic sleeve gastrectomy. NGT - normal glucose tolerance. IGT - impaired glucose tolerance. T2D - type 2 diabetes. GLP-1 - glucagon like peptide 1. OGTT - oral glucose tolerance test.				

In addition to its role in intestinal physiology, experimental studies have highlighted the effect of glicentin on glucose metabolism. Some authors addressed its variation in the context of diabetes. While a first study measured fasting glicentin concentration using a non-commercialized radioimmunoassay combined with chromatography revealed lower glicentin concentrations in type 2 diabetic patients compared to controls (*38*), others investigators did not find any significant difference using a non-commercialized sandwich ELISA in a larger cohort of patients (*39*). However, the authors found high glicentin concentrations in a diabetic patient with renal failure suggesting that glicentin may be excreted through the kidney (*39*). Given the development of new commercialized method which may improve reproducibility of circulating glicentin measurement, it would be worth investigating its variation in large cohorts of patients and different types of diabetes to evaluate its potential usefulness as non-invasive biomarker of the disease.

A recent study aimed at exploring glicentin variation in obese adolescents who had associated metabolic disorders (*42*). While no significant difference on fasting glicentin concentrations were observed between lean adolescents and adolescents with obesity and normal glucose tolerance (NGT), adolescents with obesity and impaired glucose tolerance (IGT) had significantly lower glicentin concentration at fasting state and after OGTT compared to those with obesity and NGT (*42*). Besides, the profile of glicentin secretion observed after OGTT differs between diabetic and healthy subjects and between obese adolescents with NGT, IGT or type 2 diabetes (*38*, *42*). These results suggest that glicentin concentrations may be influenced by metabolic disorders and altered glucose homeostasis rather than by the weight or the body mass index itself. This hypothesis is corroborated by the fact that no correlation was observed between body mass index and glicentin concentrations (*44*, *45*). In addition, decreased fasting glicentin concentrations was observed in patients with abnormal glucose metabolism after acute pancreatitis, confirming the link between glicentin concentration and defective glucose homeostasis (*45*). Nevertheless, the impact of glucose homeostasis on glicentin secretion may results from complex mechanisms which are not fully elucidated. Even if glucose intake stimulates glicentin secretion, no linear correlation was found between fasting glicentin and plasma glucose (*39*, *44*). Besides, in obese adolescents with IGT and type 2 diabetes, ratios of glicentin/glucagon and GLP-1/glucagon at fasting and after OGTT were lower compared to obese adolescents with NGT. This means that impaired glucose homeostasis may favour the production of pancreatically cleaved proglucagon-derived peptides (glucagon) at the expense of intestinally cleaved peptides including glicentin and GLP-1 (*42*). At last, the authors investigated if glicentin could have a predictive value of IGT in obese adolescents with normal fasting glucose and found that fasting glicentin measurement had sensitivity at 100% and specificity at 56% at a cut-off of 22.05 pmol/L, lower concentrations indicating IGT (*42*). Even if further studies are required, glicentin could potentially represent a biomarker of obesity-related metabolic disorders.

Interestingly, obese patients had lower fasting glicentin concentrations compared to control lean subjects at baseline (*44*). Moreover, a significant increase of glicentin was observed in obese patients who underwent bariatric surgery at 6 and 12 months’ post-intervention (*43*). Patients receiving a Roux-en-Y gastric bypass (RYGB), which consists in reducing the size of the stomach to a small pouch and shunting the duodenum and the proximal jejunum were compared to patients submitted to a laparoscopic sleeve gastrectomy (LSG), consisting in a longitudinal resection of the stomach. LSG is a restrictive procedure whereas RYGB associates restrictive and malabsorptive effects. The increase of glicentin post-surgery tended to be more marked after RYGB compared to LSG. Hence, bariatric surgery appears to restore, at least partly, glicentin secretion. The impact of digestive surgery on glicentin secretion has been so far poorly investigated. One study reports a higher response of glicentin secretion after OGTT in patients who had a gastrectomy compared to controls whereas no significant response was found in a patient who underwent a massive small bowel resection (*39*). Taken together, these results corroborate the findings in animal models that the distal small bowel may be a major site of glicentin production (*12*). In addition, enhanced glicentin secretion after surgery may result from early stimulation of the distal gut mucosa by nutrients as a result of rapid gastric emptying. At last, bariatric surgery has proven its efficiency to improve obesity-related metabolic disorders (*46*). As glicentin plays a role in glucose homeostasis and reversely metabolic disorders impact on its secretion, further studies are required to determine whether it could be an actor and/or a marker of metabolic improvement observed after bariatric surgery.

## Conclusion and future directions

Glicentin has been much less studied than other proglucagon-derived peptides such as GLP-1, GLP-2 or oxyntomodulin. This may be, at least partly, explained by the lack of commercialized detection methods available until recently. Glicentin is produced by L-intestinal cells from the duodenum to the rectum, with a main secretion probably located in the distal small bowel and the proximal colon. Its secretion is stimulated by food intake and experimental studies highlighted its role in intestinal growth, trophicity and motility. In addition to its paracrine function on the digestive tract, glicentin plays a role in metabolism, mainly in glucose homeostasis regulation through exerting incretin-like effects. The implication of glicentin in both intestinal physiology and glucose homeostasis points to various potential applications. Recent advances to assess its circulating variations in human offers promising perspectives to investigate its usefulness as non-invasive biomarker of various pathological states including intestinal diseases such as inflammatory pathologies or colorectal cancers, as well as metabolic disorders like diabetes or obesity. In parallel, advances in fundamental research on proglucagon-derived peptides biogenesis could provide new pathways involved in glicentin production and secretion and would be useful for a better understanding of its action and its link with other proglucagon-derived peptides. Despite its discovery a few decades ago, the field is still in its infancy and we truly believe that new technologies and further experimental and clinical studies could bring innovative perspectives to use glicentin as a biomarker and/or as a potential therapeutic target of several pathological states.
